# Treatment duration of febrile urinary tract infection: a pragmatic randomized, double-blind, placebo-controlled non-inferiority trial in men and women

**DOI:** 10.1186/s12916-017-0835-3

**Published:** 2017-04-03

**Authors:** Cees van Nieuwkoop, Willize E. van der Starre, Janneke E. Stalenhoef, Anna M. van Aartrijk, Tanny J. K. van der Reijden, Albert M. Vollaard, Nathalie M. Delfos, Jan W. van ’t Wout, Jeanet W. Blom, Ida C. Spelt, Eliane M. S. Leyten, Ted Koster, Hans C. Ablij, Martha T. van der Beek, Mirjam J. Knol, Jaap T. van Dissel

**Affiliations:** 1 Department of Internal Medicine, Haga Teaching Hospital, Els-Borst Eilersplein 245, 2545 AA, The Hague, The Netherlands; 2 Department of Infectious Diseases, Leiden University Medical Center, Leiden, The Netherlands; 3 Department of Internal Medicine, Alrijne Hospital, Leiderdorp, The Netherlands; 4 Department of Internal Medicine, Bronovo Hospital, The Hague, The Netherlands; 5 Department of Public Health and Primary Care, Leiden University Medical Center, Leiden, The Netherlands; 6 Primary Health Care Center, Wassenaar, The Netherlands; 7 Department of Internal Medicine, Medical Center Haaglanden, The Hague, The Netherlands; 8 Department of Internal Medicine, Groene Hart Hospital, Gouda, The Netherlands; 9 Department of Internal Medicine, Alrijne Hospital, Leiden, The Netherlands; 10 Department of Medical Microbiology, Leiden University Medical Center, Leiden, The Netherlands; 11 National Institute for Public Health and the Environment (RIVM), Centre for Infectious Disease Control (CIb), Bilthoven, The Netherlands

**Keywords:** Antibiotic therapy, Treatment duration, Pyelonephritis, Urinary tract infection

## Abstract

**Background:**

In adults with febrile urinary tract infection (fUTI), data on optimal treatment duration in patients other than non-pregnant women without comorbidities are lacking.

**Methods:**

A randomized placebo-controlled, double-blind, non-inferiority trial among 35 primary care centers and 7 emergency departments of regional hospitals in the Netherlands. Women and men aged ≥ 18 years with a diagnosis of fUTI were randomly assigned to receive antibiotic treatment for 7 or 14 days (the second week being ciprofloxacin 500 mg or placebo orally twice daily). Patients indicated to receive antimicrobial treatment for at least 14 days were excluded from randomization.

The primary endpoint was the clinical cure rate through the 10- to 18-day post-treatment visit with preset subgroup analysis including sex. Secondary endpoints were bacteriologic cure rate at 10–18 days post-treatment and clinical cure at 70–84 days post-treatment.

**Results:**

Of 357 patients included, 200 were eligible for randomization; 97 patients were randomly assigned to 7 days and 103 patients to 14 days of treatment. Overall, short-term clinical cure occurred in 85 (90%) patients treated for 7 days and in 94 (95%) of those treated for 14 days (difference –4.5%; 90% CI, –10.7 to 1.7; *P*_non-inferiority_ = 0.072, non-inferiority not confirmed). In women, clinical cure was 94% and 93% in those treated for 7 and 14 days, respectively (difference 0.9; 90% CI, –6.9 to 8.7, *P*_non-inferiority_ = 0.011, non-inferiority confirmed) and, in men, this was 86% versus 98% (difference –11.2; 90% CI –20.6 to –1.8, *P*_superiority_ = 0.025, inferiority confirmed).

The bacteriologic cure rate was 93% versus 97% (difference –4.3%; 90% CI, –9.7 to 1.2, *P*_non-inferiority_ = 0.041) and the long-term clinical cure rate was 92% versus 91% (difference 1.6%; 90% CI, –5.3 to 8.4; *P*_non-inferiority_ = 0.005) for 7 days versus 14 days of treatment, respectively. In the subgroups of men and women, long-term clinical cure rates met the criteria for non-inferiority, indicating there was no difference in the need for antibiotic retreatment for UTI during 70–84 days follow-up post-treatment.

**Conclusions:**

Women with fUTI can be treated successfully with antibiotics for 7 days. In men, 7 days of antibiotic treatment for fUTI is inferior to 14 days during short-term follow-up but it is non-inferior when looking at longer follow-up.

**Trial registration:**

The study was registered at ClinicalTrials.gov [NCT00809913; December 16, 2008] and trialregister.nl [NTR1583; December 19, 2008].

**Electronic supplementary material:**

The online version of this article (doi:10.1186/s12916-017-0835-3) contains supplementary material, which is available to authorized users.

## Background

In the last decade, treatment of urinary tract infections (UTIs) has become more complicated by the rising antimicrobial resistance of Enterobacteriaceae, the most common uropathogens [1]. With a scarcity of new antimicrobial classes in the development pipe-line, it is essential to develop strategies to maintain effectiveness of the available antimicrobials [2]. Therefore, among strategies to control resistance, the determination of an optimal duration of treatment is essential in addition to optimization of diagnostics to target treatment and antibiotic stewardship concerning antibiotic choice and dose. Shortening of antimicrobial therapy will lead to less selection pressure on the gut microbiome with benefits to both the individual patient as well as the ecological environment, including reduction of antibiotic resistance development [3]. Therefore, the focus upon treatment duration of common infections should be that shorter is better [4]. With respect to febrile UTI (fUTI) or acute pyelonephritis, trials upon treatment duration have usually focused on previously healthy young women and have addressed optimal treatment duration by comparing the same drug for different durations of therapy, or compared various treatment durations of different antimicrobial agents [5]. As such, recommendations upon optimal treatment duration of UTIs in men, the elderly, hospitalized patients, and patients with comorbidities or bacteremia, remain unclear [5–7].

Recently, a randomized placebo-controlled trial showed that community-acquired acute uncomplicated pyelonephritis in women of all ages can be safely and efficaciously treated with oral ciprofloxacin for 7 days [8]. Clearly, such findings need to be extended to men and patients with significant comorbidities. In the present investigator-initiated randomized trial of treatment duration, we use fUTI as the clinical syndrome of interest because this is a broadly recognized specific clinical presentation of patients. Consecutive patients with fUTI were included, including men and women with comorbidities, and treated with antibiotics for 7 or 14 days. The aims of the study were to compare clinical and bacteriological cure at both the short and long term.

## Methods

### Study design and patients

We conducted a randomized, placebo-controlled, double-blind, multicenter, non-inferiority trial; the protocol has been published previously [9].

Consecutive women and men aged 18 years or older with a presumptive diagnosis of community-acquired fUTI established by a primary care physician or on presentation at the hospital’s emergency department were screened for enrollment. Eligible patients had all of the following criteria: fever of ≥ 38.2 °C and/or a history of feeling feverish with shivering or rigors in the past 24 hours, one or more symptoms suggestive of UTI (i.e., dysuria, frequency, urgency, perineal or suprapubic pain, costovertebral tenderness, or flank pain), and positive urine nitrate test and/or pyuria (positive leucocyte esterase test or more than five leucocytes per high-power field in a centrifuged sediment). Patients enrolled were competent to provide written informed consent. Exclusion criteria for study entry were known allergy to fluoroquinolones, pregnancy or lactation, polycystic kidney disease, permanent renal replacement therapy, kidney transplantation, residence outside The Netherlands, and inability to speak or read Dutch.

Contra-indications for randomization were isolation of ciprofloxacin-resistant causal uropathogen, presence of renal abscess, metastatic infectious foci, or underlying chronic bacterial prostatitis as defined by recurrent UTI with the same uropathogen. Patients enrolled with fUTI but not randomized to trial medication, remained in the observational part of the study to assess outcome.

The study protocol was approved by the Medical Ethics Committee of the Leiden University Medical Center (protocol P08.65). In addition, the independent scientific boards of all participating hospitals assigned for local participation. The trial was registered at ClinicalTrials.gov (NCT00809913; December 16, 2008) and trialregister.nl (NTR1583; December 19, 2008).

### Randomization, antimicrobial treatment, and microbiological methods

Patients were randomized in a 1:1 ratio, stratified per center and sex, to receive either a 7-day or a 14-day regimen of antimicrobial treatment. A computer-generated randomization list, including the numbers 1 to 500, with 125 permuted blocks of four was made. The list and corresponding treatment (placebo or ciprofloxacin) was saved in an independent database with restricted access by an independent pharmacist. Randomization, and thus treatment allocation, was done once the results of the urine culture became available at the third or fourth day after inclusion. The first week of treatment was open label. In the second week, treatment was continued double-blinded, with either ciprofloxacin 500 mg or placebo orally twice daily (identical capsules for placebo and ciprofloxacin were used), according to randomization code. In inpatients, the treating physician could administer discretionary empirical intravenous antibiotics at the start of treatment according to local guidelines (in all participating centers: a β-lactam antibiotic ± aminoglycoside). These patients were switched as soon as deemed possible to open label oral ciprofloxacin (non-blinded) up to the seventh day after inclusion (equal to day of presentation with febrile UTI and start of antibiotic treatment). The decision whether to treat as outpatient or inpatient was made by the attending physician based on clinical judgment. In case the urine culture was negative or contaminated, patients were only randomized if the attending physician indicated the patient should be further treated for fUTI and no alternative diagnosis was made. Cultures were analyzed according to standard microbiological methods at local certified laboratories. For urine cultures, 10 μL of uncentrifuged urine was inoculated onto culture media. Plates were investigated for growth after 18–24 h of aerobic incubation at 37 °C. The amount of growth was assessed and scored from < 100 CFU/mL (no growth) to > 10^5^ CFU/mL.

A positive urine culture was defined as ≥ 10^4^ CFU/mL of urine in women, or ≥ 10^3^ CFU/mL of urine in men, or ≥ 10^2^ CFU/mL of urine collected during antibiotic treatment of UTI [9]. Further details on randomization, trial medication, microbiological methods, and study procedures have been previously published [9].

### Main outcome measures

The primary endpoint was the clinical cure rate through the 10- to 18-day post-treatment visit (short-term clinical cure). Clinical cure was defined as being alive with absence of fever and resolution of UTI symptoms (either absence of symptoms or at least 2 points improvement on a 0–5 point severity score scale), without additional antimicrobial therapy (for relapse of UTI). Secondary outcome measures were bacteriological cure through the 10- to 18-day post-treatment visit, clinical cure rate through the 70- to 84-day post-treatment visit (cumulative clinical cure), all-cause mortality, adverse event rate determined at 10–18 days and 70–84 days post-treatment, and rate of UTI relapses. In addition, outcome measures were analyzed as stratified by specific predefined subgroups (men, patients with complicated UTI, older age, patients with bacteremic UTI). Bacteriologic cure was defined as eradication of the study entry uropathogen with no recurrence of bacteriuria (pathogen growth < 10^4^ CFU/mL in women or < 10^3^ CFU/mL in men of a midstream urine culture combined with disappearance of leucocyturia) [10].

### Statistical analysis

The primary endpoint was analyzed on the intention-to-treat (ITT) population, including all randomized patients who received at least one dose of the study drug (on the eighth day of UTI treatment), and on the per-protocol (PP) population, including all randomized patients who had been given the study drug for a minimum of 24 hours (in case of treatment failure) or who had taken at least 80% of the study drug (in case of clinical cure).

The study sample size was calculated on the basis of a clinical cure rate of 10 percentage points lower at short-term follow-up in the 7-day treatment arm with the assumption of a 90% clinical cure rate in patients treated for 14 days [11, 12]. We adopted 10% as the margin of non-inferiority as suggested previously [13]. As we were only interested in non-inferiority and not in equivalence, the sample size calculation was based on a one-tailed alpha of 0.05. Assuming a non-inferiority margin of 0.10, 1-tailed alpha of 0.05, and a power of 0.90, the required sample size per group was 200. This implies that the 90% confidence interval of a two-tailed χ^2^ test should not cross the predefined risk difference of 10% lower clinical cure rate, or equivalently, the one-sided *P* value is less than the 0.05 significance level [14]. Interim analyses were done after randomization of 100 and 200 patients. After the second interim analysis, there was no reason to stop the trial for safety reasons. However, the principal investigators, who obviously were still blinded with respect to treatment allocation, noted that the overall cure rate was 92%. This is comparable with the results of a recently published similar trial in women comparing 73 to 83 patients treated over 7 or 14 days, respectively [8]. As we had indeed included a larger sample size of 200 patients, we estimated that our study would likely have already met the criteria for non-inferiority while still having a power of 0.80 with a type 1 error of 0.05. As we were confronted with an almost empty budget and a dropping inclusion rate after almost 5 years of participation, we considered continuation of the trial was no longer realistic and thus we decided to stop the trial at this point.

Descriptive statistics were used to describe the baseline characteristics in each arm with χ^2^ tests for binomial and categorical data and Mann–Whitney tests for continuous data. All analyses were performed using SPSS 20.0 (SPSS Inc., Chicago, IL, USA). Confidence intervals around the risk difference were calculated using Episheet (www.krothman.org) and *P* values for non-inferiority were calculated accordingly. Interaction between predefined covariables and treatment was tested by calculating a *P* value for difference in risk differences between subgroups.

## Results

**Fig. 1:**
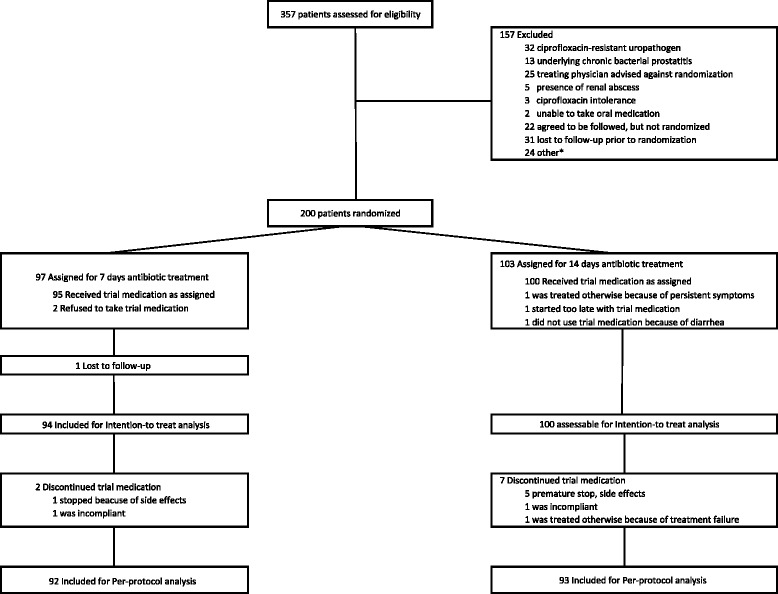
Trial profile. * concurrent medical conditions (n = 16), logistic reasons (n = 5), abroad during treatment with study medication (n = 3)

Between November 2008 and May 2013, 357 patients with a diagnosis of fUTI were enrolled into the study. Of these, 200 were randomly assigned to receive antimicrobial treatment for 7 (*n* = 97) or 14 (*n* = 103) days. Reasons for exclusion from randomization, ITT, and PP analyses are listed in Fig. 1.

Of the 157 non-randomized patients, 119 (76%) were evaluable for short-term efficacy and 116 (74%) for cumulative efficacy.

Baseline characteristics of the study population are summarized in Table 1.

**Table 1:** Baseline characteristics of 357 patients with febrile urinary tract infection

	Randomized (n = 200)		Not randomized (n = 157)	*P* value^c^
	Antibiotic treatment for 7 days (n = 97)	Antibiotic treatment for 14 days (n = 103)		
Age (years)	60 (48–72)	61 (40–73)	63 (49–75)	0.277
Male sex	44 (45%)	42 (41%)	58 (37%)	0.247
Body mass index (kg/m^2^, mean, SD)	26.3 (5.2)	25.8 (4.5)	26.1 (4.9)	0.969
Urologic history				
Indwelling urinary catheter	3 (3%)	2 (2%)	12 (8%)	0.024
Urinary tract disorder^a^	28 (29%)	28 (27%)	52 (33%)	0.296
Recurrent UTI^b^	19 (20%)	19/100 (19%)	47/147 (32%)	0.007
Comorbidity				
Diabetes mellitus	12 (12%)	17 (17%)	25 (16%)	0.709
Malignancy	3 (3%)	5 (5%)	17 (11%)	0.012
Heart failure	12 (12%)	6 (6%)	19 (12%)	0.340
Cerebrovascular disease	5 (5%)	5 (5%)	13 (8%)	0.210
Chronic renal insufficiency	3 (3%)	2 (2%)	10 (6%)	0.070
COPD	10 (10%)	11 (11%)	23 (15%)	0.236
Immunocompromised	3 (3%)	8 (8%)	14 (9%)	0.209
Signs and symptoms at presentation				
Presentation at emergency department	59 (61%)	68 (66%)	145 (92%)	<0.001
Antibiotic pretreatment	23 (24%)	29 (28%)	56 (36%)	0.048
Fever duration, hours	30 (15–48)	36 (20–60)	48 (19–96)	0.081
Dysuria	82/95 (86%)	78/102 (77%)	102/145 (70%)	0.019
Flank pain	57/96 (59%)	67/102 (66%)	91/144 (63%)	0.914
Suprapubic pain	51/96 (53%)	48/100 (48%)	72/145 (50%)	0.876
Perineal pain	4/96 (4%)	7/98 (7%)	8/140 (6%)	0.986
Shaking chills within previous 24 hours	63/97 (65%)	60/101 (59%)	102/149 (70%)	0.256
Temperature > 38 °C	66 (68%)	76 (74%)	121 (77%)	0.226
Systolic blood pressure (mm Hg, mean, SD)	132 (19)	132 (22)	129 (20)	0.324
Pulse rate (beats/minute)	93 (17)	94 (19)	97 (19)	0.360
Outpatient treatment	45 (46%)	45 (44%)	23 (15%)	<0.001
Positive urine culture	69 (71%)	68 (66%)	107 (68%)	0.944
Positive blood culture	20/88 (23%)	15/98 (15%)	45/153 (29%)	0.012
Positive urine and/or blood culture	75 (77%)	70 (68%)	118 (75%)	0.571
Initial intravenous dose(s) of antibiotics	48 (50%)	55 (53%)	133 (85%)	< 0.001

Data presented as number (%) or median (IQR)

*COPD* chronic obstructive pulmonary disease, *UTI* urinary tract infection

^a^Any functional or anatomical abnormality of urinary tract except urinary catheter

^b^Three or more UTIs in past 12 months or two or more UTIs in past 6 months

^c^Randomized (both 7 and 14 days ciprofloxacin) vs. not-randomized patients

Randomized, evaluable subjects in the two treatment arms were well matched with respect to demographic characteristics and presentation on study entry. The 157 patients who were not randomized, generally had more comorbidities and were more frequently referred to the emergency department. Additional details are listed in Additional file 1. Baseline urine cultures were performed in 341 patients (96%) (Table 2).

**Table 2:** Urine culture results at entry^a^

	Randomized		Not randomized
	Antibiotic treatment for 7 days	Antibiotic treatment for 14 days	
*Escherichia coli*	65 (68%)	65 (59%)	85 (51%)
Klebsiella spp.	2 (2%)	4 (4%)	13 (8%)
*Proteus spp.*	1 (1%)	6 (5%)	6 (4%)
*Pseudomonas aeruginosa*	–	–	2 (1%)
*Enterococcus spp.*	1 (1%)	–	8 (5%)
*Staphylococcus spp.*	–	–	1 (1%)
Other^b^	3 (3%)	3 (3%)	8 (5%)
None or contaminated culture	22 (23%)	32 (29%)	45 (27%)

Data presented as number (%). Urine culture performed in antibiotic treatment for 7 days: 91 (94%), antibiotic treatment 14 days: 100 (97%), non-randomized: 150 (96%)

^a^Some patients had multiple isolates; antibiotic treatment 7 days: *n* = 6, antibiotic treatment 14 days: *n* = 10, not randomized *n* = 17

^b^Antibiotic treatment 7 days: Proteus mirabilis (*n* = 1), Citrobacter sedlakii (*n* = 1), Citrobacter koseri (*n* = 1), Candida spp. (*n* = 2); Antibiotic treatment 14 days: Morganella morganii (*n* = 1), β-hemolytic streptococci (*n* = 2); Not randomized: Serratia marcescens (*n* = 1), β-hemolytic streptococci group B (*n* = 1), Enterobacter cloacae (*n* = 1), Streptococcus bovis (*n* = 1), Citrobacter koseri (*n* = 1), *Morganella morganii* (*n* = 1), *Proteus mirabilis* (*n* = 1), *β-hemolytic streptococci* (*n* = 1)

In 99 (28%) patients, urine culture showed either no significant bacteriuria or a mixed flora; in over half of these cases (58%), patients were pre-treated with antibiotics (group randomized to 7 days: 13 (59%); group randomized to 14 days: 20 (63%)); a similar percentage pertained to those not randomized (*n* = 23, 51%).

Blood cultures were obtained in 339 patients, of which 80 (24%) had bacteremia with growth of *E. coli* in the majority of the cases (*n* = 67, 84%).

Both treatment regimens resulted in a high clinical cure rate at short-term follow-up in the ITT population (90% vs. 95% in patients treated for 7 or 14 days, respectively) (Table 3). The difference in short-term clinical cure rate between both treatment arms was 4.5% (90% CI, –10.7 to 1.7, *P*_non-inferiority_ = 0.072). Accordingly, the criteria for non-inferiority were not met as the 90% CI exceeded the predefined non-inferiority margin of 10%. The median time to defervescence did not differ between the two groups: 2 (IQR, 1–2) days in 7-day antimicrobial treatment, 2 (IQR, 1–3) days in 14-day antimicrobial treatment. Short-term clinical cure was 85% in non-randomized patients, whereas median time to defervescence amounted to 2 (IQR, 1–3) days. Among all analysis performed, there were no significant differences between ITT and PP analysis. Therefore, within the following, only outcomes of ITT analysis are presented.

**Table 3:** Clinical and bacteriologic outcomes in the intention-to-treat and per-protocol population

	Randomized		Difference (90% CI)	Non-inferiority test *P* value	Not randomized population
	Antibiotic treatment for 7 days	Antibiotic treatment for 14 days			
Intention-to-treat population	(n = 94)	(n = 99)			
Short-term efficacy^a^	(n = 94)	(n = 99)			(n = 119)
Clinical cure^b^	85 (90.4%)	94 (94.9%)	–4.5% (–10.7 to 1.7)	0.072	101 (84.9%)
Bacteriologic cure^c^	86/93 (92.5%)	89/92 (96.7%)	–4.3% (–9.7 to 1.2)	0.041	94/109 (86.2%)
Cumulative efficacy^d^	(n = 94)	(n = 94)			(n = 116)
Clinical cure^b^	87 (92.6%)	86 (91.5%)	1.1% (–5.5 to 7.6)	0.005	88 (75.9%)
Per-protocol population	(n = 92)	(n = 92)			
Short-term efficacy^a^	(n = 92)	(n = 92)			NA
Clinical cure^b^	83 (90.2%)	87 (94.6%)	–4.3% (–10.8 to 2.1)	0.073	
Bacteriologic cure^c^	84/91 (92.3%)	83/86 (96.5%)	–4.2% (–9.9 to 1.4)	0.045	
Cumulative efficacy^d^	(n = 92)	(n = 87)			
Clinical cure^b^	85 (92.4%)	79 (90.8%)	1.6% (–5.3 to 8.4)	0.005	

Data presented as number (%), unless otherwise indicated. NA: not applicable

^a^Short-term efficacy: endpoints assessed at 10- to 18-days post-treatment visit

^b^Clinical cure: being alive with absence of fever and resolution of UTI symptoms through post-treatment visit with no additional antimicrobial therapy for a relapse of UTI prescribed

^c^Bacteriologic cure: elimination of study entry uropathogen or pathogen growth < 10^4^ CFU/mL (women) or <10^3^ CFU/mL (men) combined with disappearance of leucocyturia

^d^Cumulative efficacy: endpoint assessed at 70- to 84-days post-treatment visit

**Fig. 2:**
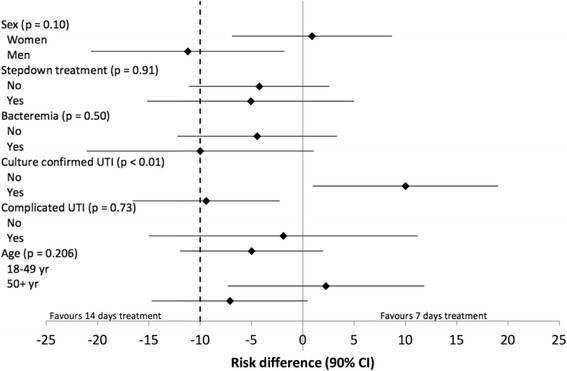
Difference in clinical cure rates (10- to 18-days post-treatment) of febrile UTI treated for 7 days versus 14 days in specific subgroups. Stepdown treatment implies initial empiric intravenous antibiotic treatment. *UTI* urinary tract infection; *CI* confidence interval. *P* values represent test for interaction. Data presented from intention to treat analysis

Short-term clinical cure rates were analyzed in preset subgroups of patients. In women, short-term clinical cures for the 7- and 14-day arms were 47 of 50 (94%) versus 54 of 58 (93%), respectively. The difference in cure rate was 0.9% (90% CI, –6.9 to 8.7, *P*_non-inferiority_ = 0.011, non-inferiority confirmed). In men, clinical cure rates differed significantly between those treated for 7 or 14 days (38 of 44; 86% vs. 40 of 41; 98%) (Fig. 2). The difference in cure rate was –11.2 (90% CI, –20.6 to –1.8, *P*_non-inferiority_ = 0.417, *P*_superiority 2-sided_ = 0.050, superiority of 14-days treatment confirmed). The large difference in risk differences for age was predominantly determined by men. Therefore, and for explorative reasons, an additional subgroup analysis was also performed within the group of men and women. The results are presented in Additional file 1: Figure S1 and Figure S2, respectively. For stepdown treatment, bacteremia, and complicated UTI, the risk differences were similar between the subgroups and in all subgroups, non-inferiority was not shown.

For cumulative clinical cure rate (70 to 84 days post-treatment), 94 patients were evaluable in each treatment arm. Clinical cure rates were high: 93% vs. 91% in patients treated for 7 or 14 days; difference 1.1% (90% CI, –5.5 to 7.6, *P*_non-inferiority_ = 0.005, non-inferiority confirmed) (Table 3). Criteria for non-inferiority for cumulative clinical cure rate were also met for the subgroups men and women (Additional file 1: Table S1).

Both treatment regimens post-randomization, were well tolerated with no differences in side effects (Additional file 1).

Post-treatment urine cultures (at days 28–32) were obtained in 93 of 94 (99%) patients assigned to 7 days, in 92 of 99 (93%) patients assigned to 14 days of antimicrobial treatment, and in 109 of 119 (92%) non-randomized patients, with the short-term follow-up visit. Bacteriologic cure was 91% in the 7-day treatment arm, 97% in patients treated for 14 days, and 86% in non-randomized patients (Table 3). More details on clinical and microbiological outcomes are listed in Additional file 1.

## Discussion

Our findings show that community-acquired fUTI can be safely and efficaciously treated with antimicrobial treatment for 7 days in women as it is non-inferior to 14 days of therapy. However, in men with fUTI, the 7-day treatment was significantly inferior to 14 days of treatment.

The main strength of this trial is its pragmatic nature reflecting daily clinical practice with the inclusion of consecutive patients with fUTI, both men and women, irrespective of age and underlying medical conditions, with the notable exception of those with severe kidney disease, antibiotic allergy, and pregnancy. Several hospitals were involved, including a referral university hospital, and general practitioners, who enrolled about one fourth of our patients. Therefore, patients recruited into the study are considered representative of individuals with acute community-acquired fUTI, encompassing acute pyelonephritis, prostatitis, and the urosepsis syndrome. Over 55% of patients were initially hospitalized because of fUTI, likely because of presumed urosepsis syndrome, and a relative high number of patients had bacteremia. Of note, the findings hold for both the ITT and the PP analyses, underlying the high compliance by patients randomized with respect to the treatment protocol and precluding that poor study procedures may have concealed differences in patient management. Finally, the clinical cure rate at 90 days after initial presentation with fUTI was evaluable in 188 (94%) patients and, though characterized as a secondary outcome measure, for the whole group as well as the subgroups of men and women, they all met the criteria for non-inferiority.

There are, however, also some limitations. First of all, the diagnosis of fUTI was not confirmed by cultures for all patients. Nevertheless, it should be noted that the attending physicians still made a clinical diagnosis of fUTI is such cases, and no alternative diagnosis for fever or urinary tract symptoms was made. Secondly, our study lacks statistical power to draw confident conclusions on the various subgroups because of the limited number of patients enrolled. However, it should be noted that all subgroups analyzed were predefined in the study protocol. Finally, it should be noted that several patient categories (e.g., ciprofloxacin-resistant uropathogen, renal transplant, pregnancy, indication for antimicrobial treatment for at least 14 days) were excluded from randomization. Thus, our findings might not be generalizable to all patients with fUTI.

Our findings extend recent findings of a highly similar controlled randomized Swedish study performed in women with acute pyelonephritis, showing non-inferiority of 7- to 14-days of antimicrobial treatment [8]. Compared to our study, their patient group was younger, had less comorbidities, and fewer had complicated UTIs.

In men, our results indicate an increase in the rate of clinical and bacteriological treatment failure after the 7-day treatment as compared to 14 days. Likely, though chronic bacterial prostatitis was an exclusion criterion, this is due to prostatic involvement of the infection as this is known to be a cause of recurrent UTI, even after appropriate antimicrobial treatment [15]. There is a lack of studies on optimal treatment duration of fUTI in men [5]. One study directly compared different treatment duration in an open, prospective, and randomized trial in 72 men with community-acquired fUTI, showing similar bacteriological cure rates with ciprofloxacin 500 mg orally twice daily for either 2 or 4 weeks [16]. Similarly, a randomized, double-blind trial in Sweden lent support for the efficacy of 14-day treatment with fluoroquinolones in men [17]. Taken together, the studies confirm that, at present, a 14-day treatment regimen of fluoroquinolones is the minimum period necessary for optimal therapy of fUTI in men. Recently, however, a retrospective analysis of a large database of male veterans indicated that more than 7 days of antibiotic treatment (the vast majority being treated with ciprofloxacin) was not associated with a reduction of UTI recurrence [18]. In addition, this study showed that treatment with β-lactams was associated with a higher risk of recurrence as compared to fluoroquinolone treatment. Furthermore, they showed that UTI recurrence was independently associated with comorbidities and age. As in our study about half of the patients were initially treated with a β-lactam intravenously, implying less penetration into the prostrate [19], this may have influenced our results and may possibly explain the larger difference in cure rates within the subgroup of men with stepdown treatment. Interestingly, in line with this, we found no significant difference in men who were solely treated with ciprofloxacin, whereas in men aged less than 50 years, there was a similar cure rate with antibiotic treatment for 7 or 14 days.

Nevertheless, it should be noted that there were similar clinical cure rates between 7 and 14 days of treatment during longer follow-up (70–84 days post-treatment) and this holds both for women and men. In others words, the need for additional antibiotic UTI treatment during longer follow-up is similar irrespective of whether the initial treatment of fUTI was 7 or 14 days. Given the principles of antimicrobial stewardship, this is an interesting finding because, even in men with fUTI, this might be an argument to treat them for 7 days. Indeed, our study indicates that a further study upon treatment duration of men with fUTI should be performed, including outcome measures being set at 3 months or even longer instead of the traditional 2–6 weeks.

Given the consistency of our findings and those of the recent study in Sweden [8], we conclude that women with fUTI, irrespective of disease severity and comorbidities, can be treated orally with 7 days of adequately dosed fluoroquinolones. Ciprofloxacin was chosen as treatment because of its reliable intestinal resorption and bioavailability, and excellent antimicrobial activity against a broad spectrum of susceptible gram-negative uropathogens, making it a drug of choice in both outpatient as well as hospital settings. As a surplus, activity against perineum and vagina colonizing Enterobacteriaceae may help to prevent early recurrences [20]. An important concern has been the rise of ciprofloxacin resistance in the community, i.e., up to 15% of Enterobacteriaceae are currently resistant in The Netherlands, that may preclude the use of fluoroquinolones as first-choice empiric oral treatment of fUTI. Of great concern, in other countries, this figure has been reported as high as 40–50% [21, 22]. In countries with concurrent high rates of trimethoprim-sulfamethoxazole resistance in Enterobacteriaceae, there may be no oral antibiotic option left for general practitioners to treat fUTI at home, raising healthcare costs. These findings underscore the importance of controlling antimicrobial resistance through antibiotic stewardship, including the administration of antibiotics with optimal duration [4, 23].

## Conclusions

Women with fUTI can be successfully treated with antibiotics for 7 days, including those who initially were treated with β-lactams intravenously. In men, 7 days of antibiotic treatment for fUTI is inferior to 14 days when looking at short-term clinical cure. During long-term follow-up, even in men, 7 days of antibiotic treatment is non-inferior to 14 days.

Is should be considered that the primary outcome measures on future trials on antibiotic treatment duration of fUTI in men, should be set at 3 months instead of the traditional 2 weeks.

## Additional file

Additional file 1: Additional baseline characteristics, clinical outcomes and microbiological outcomes. (DOCX 443 kb)

## Abbreviations

CI: Confidence interval
fUTI: Febrile urinary tract infection
ITT: Intention to treat
PP: Per protocol
UTI: Urinary tract infection
