# Supplementary material for: Treatment duration of febrile urinary tract infection: a pragmatic randomized, double-blind, placebo-controlled non-inferiority trial in men and women
Source: BMC Med. 2017 Apr 3;15:70. doi: 10.1186/s12916-017-0835-3 (PMC5376681; doi:10.1186/s12916-017-0835-3)

**Additional file 1**

**Baseline characteristics**

In the 7-day treatment arm, 23 (24%) patients had been pretreated for presumptive UTI with: norfloxacin (n=1, 4%), nitrofurantoin (n=5, 22%), trimethoprim ± sulfamethoxazole (n=3, 13%), amoxicillin ± clavulanic acid (n=12, 52%), fosfomycin (n=1, 4%) and others (n=1, 4%). Of those randomized to 14 days antimicrobial treatment, 29 (28%) had been pretreated with ciprofloxacin (n=3, 10%), norfloxacin (n=1, 3%), nitrofurantoin (n=6, 21%), trimethoprim ± sulfamethoxazole (n=7, 24%), amoxicillin ± clavulanic acid (n=8, 28%), others (n=3, 10%) and unknown (n=1, 3%). In the non-randomized group, 56 (36%) had been pretreated with ciprofloxacin (n=8, 14%), norfloxacin (n=2, 6%), nitrofurantoin (n=8, 14%), trimethoprim ± sulfamethoxazole (n=7, 13%), amoxicillin ± clavulanic acid (n=21, 38%), fosfomycin (n=1, 2%), others (n=4, 7%) and unknown (n=5, 9%).

About half of the patients were initially treated with intravenous antibiotics. This did not differ between the two treatment arms: in the 7-day treatment arm, 48 (50%) patients (cefuroxime n=21, 44%; cefuroxime + gentamicin n=22, 46%; other n=5, 10%) and in the 14-day treatment arm, 55 (53%) patients (cefuroxime (n=32, 58%), cefuroxime ± gentamicin (n=20, 36%), ciprofloxacin i.v. (n=1, 2%) and other antibiotics (n=2, 4%)). In the non-randomized group, 133 (85%) patients had initial dose(s) of intravenous antibiotics, i.e., cefuroxime (n=61, 46%), cefuroxime ± gentamicin (n=49, 37%), ciprofloxacin (n=4, 3%) and other (n=18, 14%). Of note, the median time till switch from intravenous to oral antibiotics was 3 days (IQR 2-4), and did not differ between the groups.

**Clinical outcome**

During short-term follow-up, nine patients assigned to antibiotic treatment for 7 days, had a clinical recurrence. Three patients had an episode of (afebrile) acute cystitis at day 17, 18 and 20, whereas six patients had an additional episode of fUTI at day 9, 14, 15, 17, 20 and 26 after treatment. Among patients assigned to 14 days of antibiotic treatment, one patient had an acute cystitis at day 30 and four patients had recurrent fUTI at day 8, 9, 19 and 20.

During late follow-up, seven patients assigned to 7 days had a clinical recurrence. Six patients had an episode of (afebrile) acute cystitis at day 38, 40, 56, 63, 64 and 83 and one patient had an additional episode of fUTI. Among patients assigned to 14 days, seven patients had an (afebrile) acute cystitis at day 40, 44, 71 and 77 (n = 3, day unknown) and one patients had recurrent signs of fUTI at day 90.

One patient assigned to antibiotic treatment for 7 days was readmitted at day 9 because of treatment failure, and was treated intravenously with cefuroxime followed by oral ciprofloxacin for 14 days, now with good clinical response. None of the patients assigned to the 14-days treatment arm were readmitted because of treatment failure.

For the primary outcome measure, additional subgroup analysis within the group of men and women are outlined in Figure A and B, respectively. There were no apparent differences within all subgroups except for men without culture confirmed UTI and men < 50 years old, in which 7 days of antibiotic treatment is non-inferior to 14 days (see Figure A). 12 patients were aged < 50 years. The clinical cure rate (10-to 18-days post treatment) in these men was 100% for both the 7-day and 14-day treatment arm (see Figure A). For men aged ≥ 50 years, the clinical cure rate was 85% versus 97% for 7 days versus 14 days of treatment (p_superiority_ = 0.027). For women aged < 50 years the clinical cure rates were 95% and 92 % for the 7-day and 14-day treatment arm respectively. For women aged ≥ 50 years this was 93% and 94%. Other outcome measures for men and women are outlined in Table A.

During the study period, no patients treated for 7 days died. One patient, a 84-year old man assigned to 14 days antimicrobial treatment, died on day 92 due to pneumonia and sepsis. Five non-randomized patients died during follow-up due to concurrent medical problems. None of the patients developed pyonephrosis or renal stones requiring additional drainage. Ten patients were temporarily treated with a bladder catheter; 2 patients in the 14-day treatment arm and 8 patients in the 7-day treatment arm.

With respect to side effects, one patient who received placebo, discontinued the trial drug because of mucosal candida infection (day 2 after start placebo). Five patients on ciprofloxacin discontinued trial drug because of itching exanthema (n=2, both on day 3, i.e., day 10 of treatment) or feeling tired (n=3; day 1,3 and 5). During trial drug period, patients reported the following adverse events in the 7 versus 14 days treatment arm: nausea (7% vs 4%), vomiting (2% vs 1%), diarrhea (3% vs 2%), headache (16% vs 4%), dizziness (10% vs 9%), itching exanthema or rash (4% vs 4%) and myalgia (10% vs 12%).

**Microbiological outcome**

In the group assigned to 7 days of ciprofloxacin, seven patients had asymptomatic bacteriuria at short-term follow-up (five with *E. coli*, one with *Klebsiella oxytoca* and one with *Enterococcus faecalis*). Three patients treated with ciprofloxacin for 14 days had asymptomatic bacteriuria at short-term follow-up (one with *E. coli*, one with *E. faecalis* and one with coculture of *E. faecalis* and *S. aureus*). Fifteen non-randomized patients had asymptomatic bacteriuria at short-term visit: seven with *E. coli*, one with *E. coli* and *E. faecalis*, one with *Klebsiella* spp and *S. saprophyticus*, one with *Proteus* spp, two with *E. faecalis*, one with *E. faecalis* and *P. aeruginosa*, one with *P. aeruginosa* and one with *Enterobacter cloacae*.

**Figure A. Difference in clinical cure rates (10- to 18-days post-treatment) of febrile UTI treated for 7 days versus 14 days in specific male subgroups.**

Stepdown treatment implies initial empiric intravenous antibiotic treatment. UTI: urinary tract infection; CI: confidence interval.


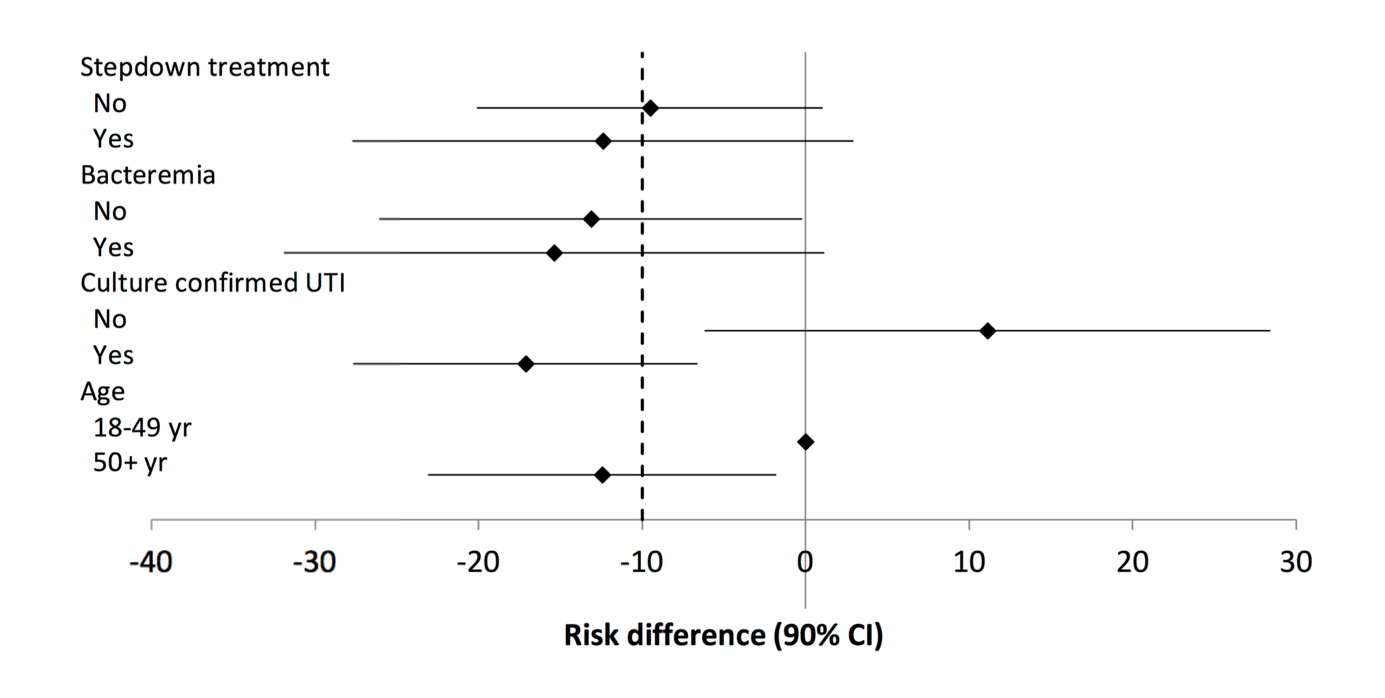


**Figure B. Difference in clinical cure rates (10- to 18-days post-treatment) of febrile UTI treated for 7 days versus 14 days in specific female subgroups.**

Stepdown treatment implies initial empiric intravenous antibiotic treatment. UTI: urinary tract infection; CI: confidence interval.


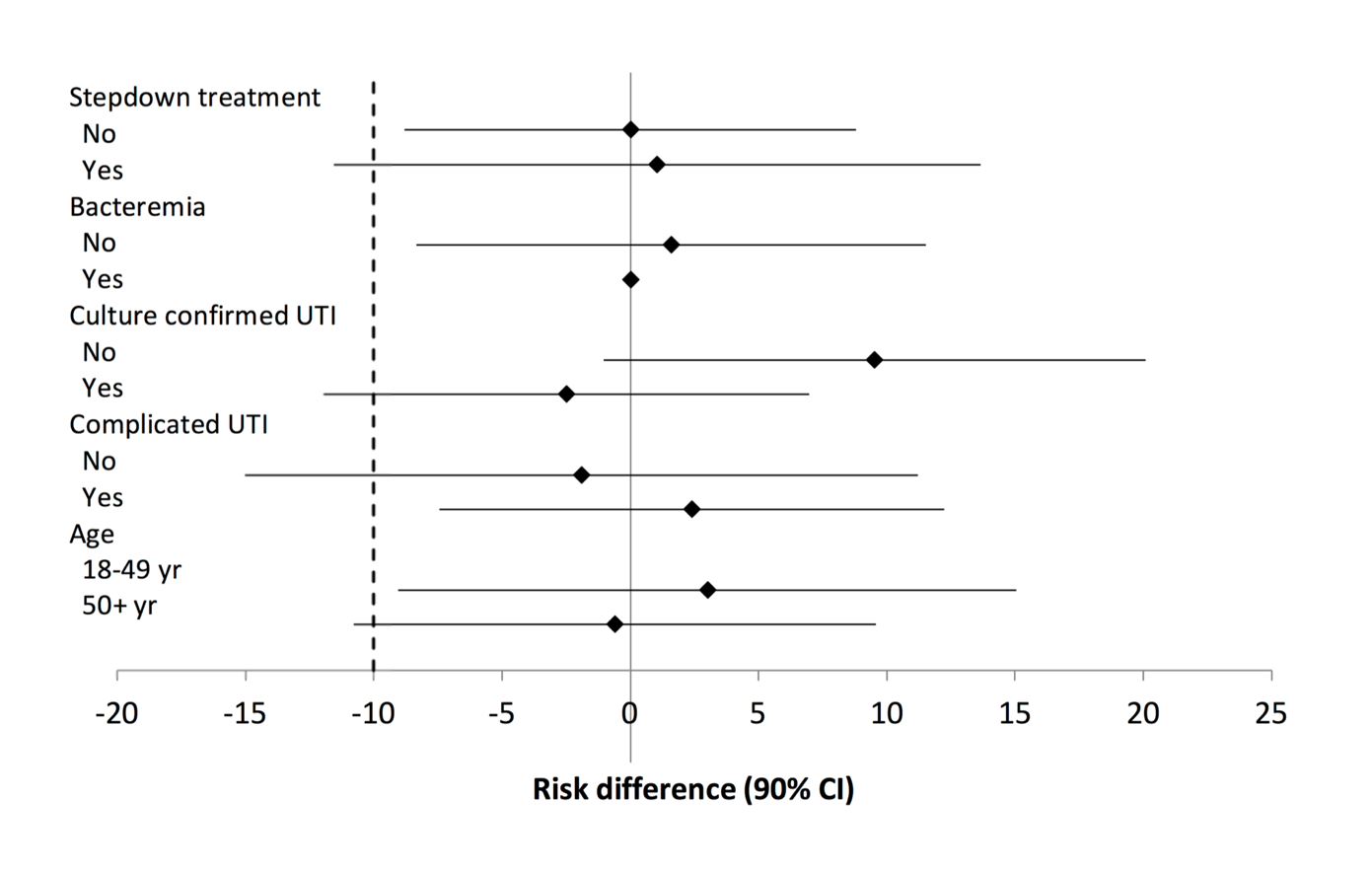


**Table A. Difference in outcome measure of 7 days antimicrobial treatment for febrile UTI versus 14 days in men and women.**


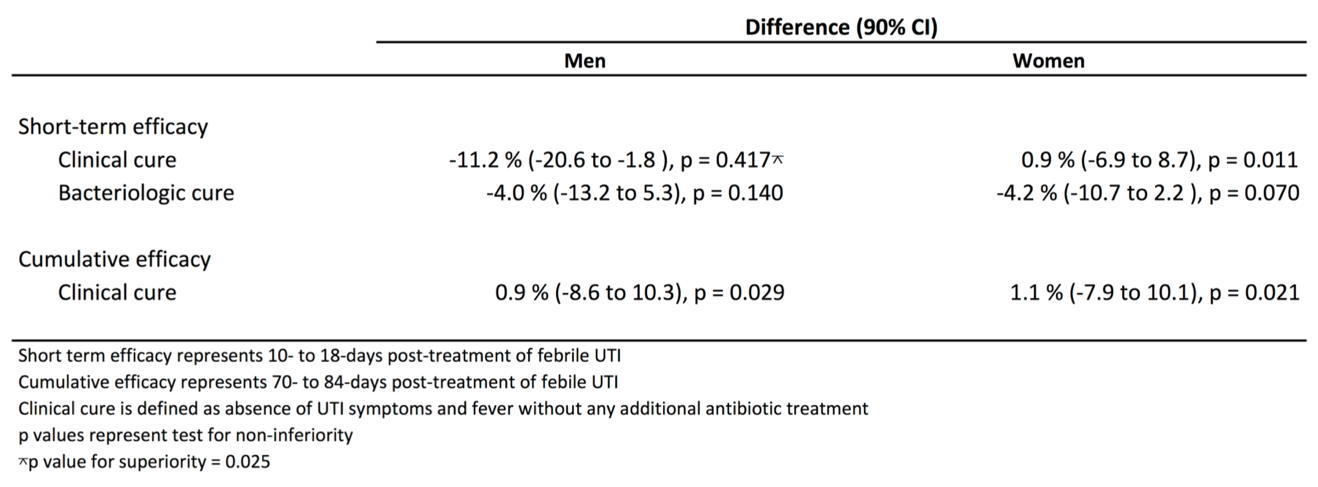

Supplement: Additional file 1: — Additional baseline characteristics, clinical outcomes and microbiological outcomes. (DOCX 443 kb) [file 12916_2017_835_MOESM1_ESM.docx]
